# Supplementary material for: Gender-Related Differences in BMP Expression and Adult Hippocampal Neurogenesis within Joint-Hippocampal Axis in a Rat Model of Rheumatoid Arthritis
Source: Int J Mol Sci. 2021 Nov 10;22(22):12163. doi: 10.3390/ijms222212163 (PMC8620092; doi:10.3390/ijms222212163)
Supplement: Supplementary file 1 [file ijms-22-12163-s001.zip › ijms-1435499-supplementary.pdf]

Co-expression of DCX with BMP-4 and Noggin in the hippocampus/dentate gyrus

Immunofluorescent analyses confirmed higher co-expression of BMP-4 with DCX in the hippocampus/dentate gyrus in male control rats and at PIA peak in regard to female control rats (Figure S1: p=0.000, p < 0.001). DCX and Noggin co-expression was confirmed at higher levels in male control rats (Figure S1: p < 0.001). These results may imply a higher level of protection for adult neurogenesis in general in male rats.

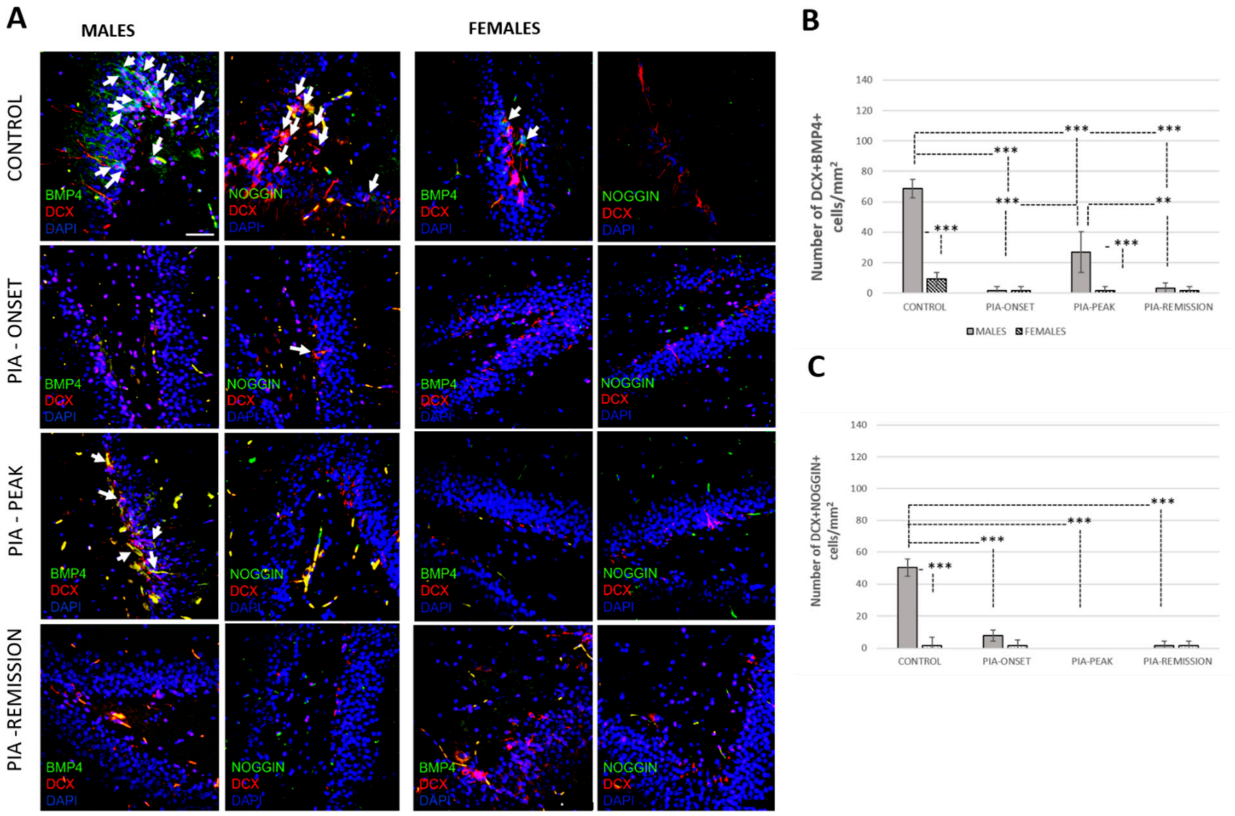

Figure S1. Co-expression of DCX with BMP-4 and Noggin in the hippocampus/dentate gyrus. (A) Representative immunofluorescent pictures show co-expression of DCX with BMP-4 and Noggin in paraffin-embedded sections of brain tissue obtained from male and female DA rats: control (treated with saline); PIA onset (between 9th and 12th day after induction); PIA peak (between 16th and 20th day after induction), PIA remission (between 20th and 25th day after induction). (B,C) The number of DCX^+ $BMP\text{-}4^+$ cells, and DCX^+ $Noggin^+$ cells per mm^2 were manually counted in regions of interest (8 ROI/4 µm slide x 3 slides/rat x 6 rats/group; N = 24). One–way ANOVA followed by the post hoc Scheffé test: ** $p < 0.01$ and *** $p < 0.001$. Scale bars indicate 50 µm.

Co-expression of DCX with BMP-7 and Gremlin in the hippocampus/dentate gyrus

Immunofluorescent analyses confirmed higher co-expression of BMP-7 with DCX in the hippocampus/dentate gyrus in male control rats and at PIA peak in regard to female control rats (Figure S2: $p < 0.001$, $p=0.04$). Rats of both genders show an increase in BMP-7 expression in the remission phase (Figure S2B). Furthermore, co-expression of Gremlin with DCX was confirmed at higher levels at PIA peak in male rats (Figure S2: $p < 0.001$), which could provide better protection for adult neurogenesis.

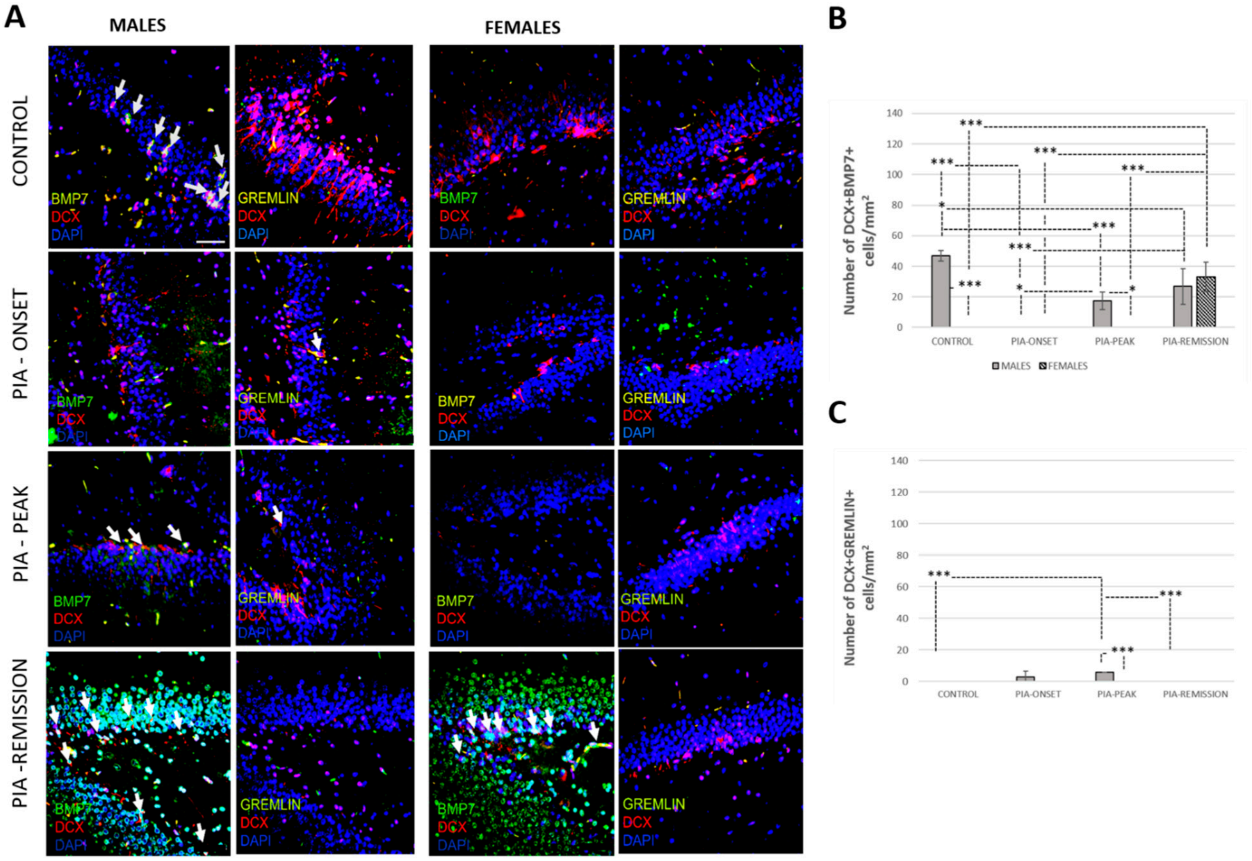

Figure S2. Co-expression of DCX with BMP-7 and Gremlin in the hippocampus/dentate gyrus. (A) Representative immunofluorescent pictures show co-expression of DCX with BMP-7 and Gremlin in paraffin-embedded sections of brain tissue obtained from male and female DA rats: control (treated with saline); PIA onset (between 9th and 12th day after induction); PIA peak (between 16th and 20th day after induction), PIA remission (between 20th and 25th day after induction). (B,C) The number of DCX^{+} $BMP\text{-}7^{+}$ cells, and DCX^{+} $Gremlin^{+}$ cells per mm^2 were manually counted in regions of interest (8 ROI/4 μm slide x 3 slides/rat x 6 rats/group; N = 24). One–way ANOVA followed by the post hoc Scheffé test: * $p < 0.05$ and *** $p < 0.001$. Scale bars indicate 50 μm.

Male rats express a higher level of hippocampal/dentate gyrus BMP-4 than female rats

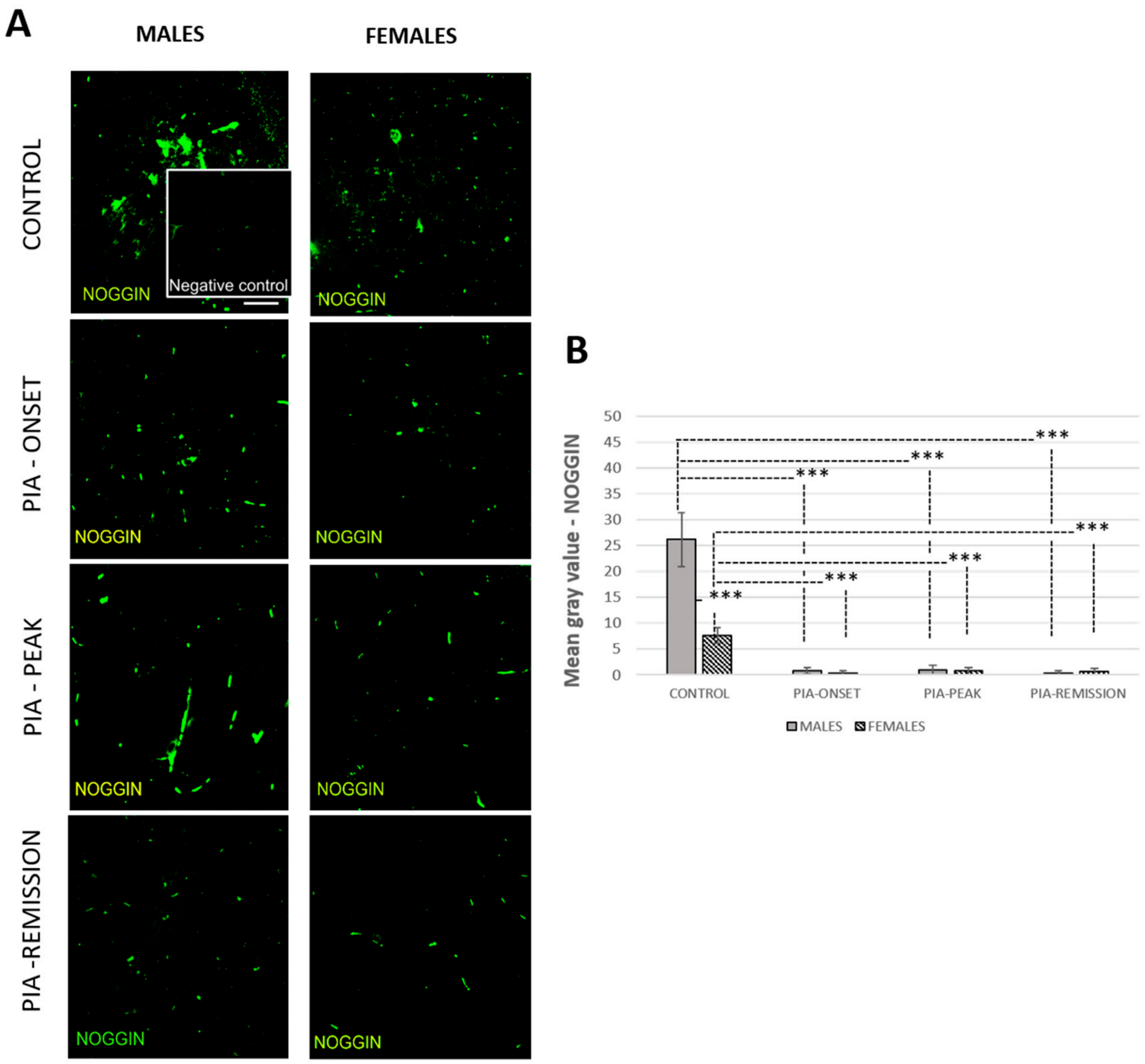

Figure S3. Pristane-induced arthritis downregulates Noggin expression in the hippocampus/dentate gyrus in DA rats. (A) Representative immunofluorescent pictures show staining with anti-Noggin antibody in paraffin-embedded sections of brain tissue obtained from male and female DA rats: control (treated with saline); PIA onset (between 9th and 12th day after induction); PIA peak (between 16th and 20th day after induction); PIA remission (between 20th and 25th day after induction). (B) Noggin immunoreactivity in the hippocampus/dentate gyrus. The immunofluorescent signal quantification was performed using Cell F v3.1 software analysis on 12 regions of interest (3 slides/rat × 6 animals/group). Values are expressed as mean grey value ± SD (N = 24). One-way ANOVA followed by the post hoc Scheffé test: *** $p < 0.001$. Insert shows staining in a slide incubated without primary anti-Noggin antibody (negative control). Scale bars indicate 50 μm.

Male rats express a higher level of hippocampal/dentate gyrus Gremlin than female rats

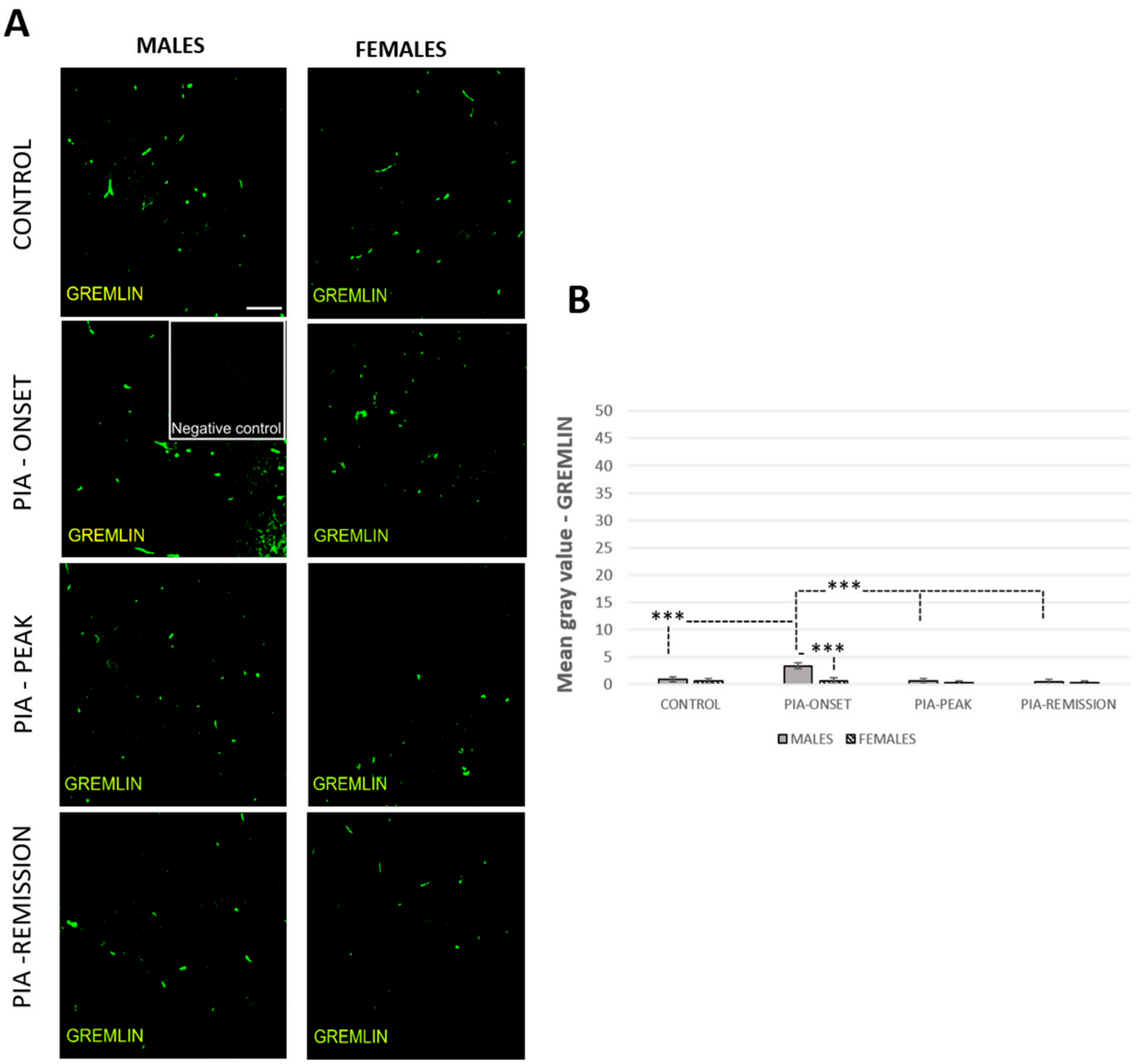

Figure S4. Pristane-induced arthritis upregulates Gremlin expression in the hippocampus/dentate gyrus in male rats. (A) Representative immunofluorescent pictures show staining with anti-Gremlin antibody in paraffin-embedded sections of brain tissue obtained from male and female DA rats: control (treated with saline); PIA onset (between 9th and 12th day after induction); PIA peak (between 16th and 20th day after induction); PIA remission (between 20th and 25th day after induction). (B) Gremlin immunoreactivity in the hippocampus/dentate gyrus. The immunofluorescent signal quantification was performed using Cell F v3.1 software analysis on 12 regions of interest (3 slides/rat × 6 animals/group). Values are expressed as mean grey value ± SD (N = 24). One-way ANOVA followed by the post hoc Scheffé test: *** $p < 0.001$. Insert shows staining in a slide incubated without primary anti-Gremlin antibody (negative control). Scale bars indicate 50 μm.
